# Whole-Genome Sequencing and Bioinformatics as Pertinent Tools to Support *Helicobacteracae* Taxonomy, Based on Three Strains Suspected to Belong to Novel *Helicobacter* Species

**DOI:** 10.3389/fmicb.2019.02820

**Published:** 2019-12-06

**Authors:** Elvire Berthenet, Lucie Bénéjat, Armelle Ménard, Christine Varon, Sabrina Lacomme, Etienne Gontier, Josette Raymond, Ouahiba Boussaba, Olivier Toulza, Astrid Ducournau, Alice Buissonnière, Alban Giese, Francis Megraud, Emilie Bessède, Quentin Jehanne, Philippe Lehours

**Affiliations:** ^1^French National Reference Center for Campylobacters and Helicobacters, Bordeaux, France; ^2^Univ. Bordeaux, INSERM, Bordeaux Research in Translational Oncology, BaRITOn, U1053, Bordeaux, France; ^3^CNRS, INSERM, Bordeaux Imaging Center UMS 3420 – US4, Pôle d’Imagerie Électronique, Bordeaux, France; ^4^Bacteriology, Cochin Hospital, Institut Pasteur, University of Paris-Descartes, Paris, France; ^5^Clinique Vétérinaire AQUIVET, Eysines, France

**Keywords:** whole-genome sequencing, novel species, *Helicobacter* genus, taxonomy, *gyrA*

## Abstract

The present study describes three putative novel species received at the French National Reference Center for Campylobacters & Helicobacters (CNRCH). The CNRCH 2005/566H strain was isolated in 2005 from the feces of a patient with a hepatocellular carcinoma and gastroenteritis. Strain 48519 was isolated in 2017 from the blood of a male patient suffering from a bacteremia. Strain Cn23e was isolated from a gastric biopsy from a dog suffering from chronic gastritis. Biochemical and growth characteristics and electron microscopy for these three strains were studied. Their genomes were also sequenced. *gyrA* based phylogeny built with 72 nucleotide sequences placed CNRCH 2005/566H among the unsheathed enterohepatic helicobacters, close to *Helicobacter valdiviensis*; strain 48519 among the sheathed enterohepatic helicobacters, close to *Helicobacter cinaedi*; and strain Cn23e among gastric helicobacters, close to *Helicobacter felis*. 16S rRNA gene phylogeny showed similar results, but with weak discriminant strength. Average nucleotide identity and *in silico* DNA–DNA hybridization analyses revealed that CNRCH 2005/566H and 48519 strains belong to new putative species, but confirmed that Cn23e corresponds to *H. felis*. Cn23e was able to infect C57BL6 mice and to induce gastric inflammation. The genomics data, together with their different morphological and biochemical characteristics, revealed that these two strains represent novel *Helicobacter* species. We propose the following names: ‘*Helicobacter burdigaliensis*,’ with the type strain CNRCH 2005/566H ( =CECT 8850 =CIP 111660), and ‘*Helicobacter labetoulli*,’ with the type strain 48519 ( =CCUG 73475 =CIP 1111659). This study highlights that the diversity of the *Helicobacteraceae* family remains to be fully explored.

## Introduction

To date, the *Helicobacter* genus is comprised of 41 validated species^1^. The type species for the *Helicobacter* genus is *Helicobacter pylori*, well-known for its link with gastritis, peptic ulcer disease and gastric cancer in humans (Malfertheiner et al., 2017). This species was the first to be described for this genus, by Marshall and Warren (1984). It was originally named *Campylobacter pyloridis*. Species belonging to the *Helicobacter* genus are curved, spiral or fusiform rods with size ranging from 1.5 to 10 μm long and 0.2 to 1.2 μm wide. When cultured for too long, they often become coccoidal. They are non-spore-forming and Gram negative and show flagellar activity (On et al., 2015).

The *Helicobacter* genus encompasses a large variety of species (De Witte et al., 2016; Smet et al., 2018). Two species categories are based on the niche occupied: gastric and enterohepatic. All known gastric helicobacters possess sheathed flagella, but enterohepatic species can be divided into sheathed and unsheathed subtypes (Solnick and Vandamme, 2001). Some species are host-specific while others can colonize different hosts. *Helicobacter* species are able to colonize alternative sites of the digestive tract in various hosts (mammals, birds, reptiles) (Dewhirst et al., 1994; Collado et al., 2014).

16S rRNA sequencing, a powerful tool for taxonomy, was shown to be unreliable for the identification of *Helicobacter* species as well as for other bacteria (Fox et al., 1992; Vandamme et al., 1996; Jalava et al., 1997; Dewhirst et al., 2005), in part due to horizontal gene transfers. Therefore, *gyrA* based phylogeny is currently the preferred method, to take into account the variability in the *Helicobacter* genus (Ménard et al., 2016).

The French National Reference Center for Campylobacters & Helicobacters (CNRCH) collects strains of *Campylobacter* and *Helicobacter* related species sent by private clinical laboratories and public hospitals. In addition to this routine activity, the CNRCH sometimes receives strains that collaborators failed to identify. In recent years, three helicobacter isolates were suspected to belong to novel species. These strains were characterized in the laboratory and their genomes were sequenced. We propose that two of them correspond to new putative species.

## Materials and Methods

### Bacterial Strain Isolation

Culture of isolates was performed on trypticase soy agar (Difco, Becton Dickinson, Le Pont-de-Claix, France) enriched with 5% horse blood and/or an in-house Mueller Hinton medium enriched with 10% sheep blood (Thermo-Fisher Scientific, Waltham, MA, United States) and red blood cell extract (Biorad, Hercules, CA, United States) (MH10%) for 24 to 48 h at 30, 35, 37, or 42°C under microaerobic conditions. Cultures were incubated in jars using an Anoxomat microprocessor (Mart Microbiology B.V., Lichtenvoorde, Netherlands) which creates an atmosphere of 80–90% N_2_, 5–10% CO_2_, and 5–10% H_2_. Single colonies for each of the three strains were conserved at −80°C in an in-house brucella broth with 25% glycerol.

The strain CNRCH 2005/566H has been assigned to the Collection de l’Institut Pasteur (CIP) and the Spanish Type Culture Collection (CECT) under the identification numbers 11160 and 8850, respectively. The strain 48519 has been assigned to the CIP and Culture Collection of Gothenburg University (CCUG) under the identification numbers 111659 and 73475, respectively.

### Biochemical Characterization

Enzymatic activities were assessed by using the API^®^ Campy strip (bioMérieux, Marcy-l’Étoile, France). The presence of catalase and oxidase was investigated. A disk diffusion method was used to assess the susceptibility to nalidixic acid (30 μg) and cephalothin (30 μg) Antimicrobial susceptibility testing was performed according to the European Committee for Antimicrobial Susceptibility Testing (EUCAST) recommendations on in-house Mueller-Hinton agar supplemented with 5% defibrinated sheep blood (Thermo-Fisher Scientific) (MH-F) and 20 mg/L of β-nicotinamide adenine dinucleotide (Sigma Aldrich, Merck, Darmstadt, Germany), under the same atmosphere and temperature conditions, as previously described (Sifré et al., 2015). MICs were determined for each isolate with E-test^®^ strips (bioMérieux). For levofloxacin, clarithromycin, rifampicin, amoxicillin, tetracycline, and metronidazole the cut-offs of the “Comité de l’antibiogramme de la Société Française de Microbiologie” (CA-SFM) (V.2.0. May 2019) were employed^2^. The reference strain *H. pylori* CCUG 17874 was used as a quality control strain. Strains were stored at −80°C in brucella broth supplemented with 25% glycerol.

### Imaging

The morphology, cell size, and presence of flagella were determined by transmission electron microscopy. Bacteria were scraped and introduced into a fixative solution of 2.5% glutaraldehyde in 0.1M cacodylate buffer (pH 7.4) and incubated for 1 h at room temperature. After centrifugation for 3 min at 5,000 rpm, pellets were suspended in 500 μL of 0.1M cacodylate buffer (pH 7.4). A volume of 10 μL of bacterial suspension was adsorbed on carbon grids with negative ionization (Delta Microscopy, Toulouse, France) and negatively stained with a nano-tungsten solution. Grids were examined with a transmission electron microscope (Talos F200S G2, Thermo Fisher, Eindhoven, Netherlands) at 200 kV, equipped with a ONE VIEW camera (Gatan, Paris, France).

### Genome Sequencing and Annotation

After bacterial culture, colonies were resuspended in 500 μL of water and bacterial pellets were digested using MagNA Pure 96 DNA Bacterial Lysis Buffer and proteinase K. DNA extraction was performed on a MagNA Pure 96 System (Roche Diagnostics, Penzberg, Germany) using the MagNA Pure 96 DNA and Viral NA SV Kit. Quantification and purity checks (260/280 and 260/230 ratios) were performed using NanoDrop (Thermo Scientific, Waltham, MA, United States) before external sequencing by Helixio (Saint-Beauzire, France^3^). Qubit quantification was carried out prior to sequencing. Library preparations were made using 1 ng of DNA and the Nextera XT DNA Library Preparation Kit (Illumina, Inc., San Diego, CA, United States) and validation of the libraries was performed on a bioanalyzer with the High Sensitivity DNA Assay kit (Agilent, Santa Clara, CA, United States) in order to obtain sizes ranging from 250 to 1,500 base pairs (bp). Paired-end sequencing was then performed on a NextSeq500 (Illumina). Quality was controlled using FastQC v0.11.3 (Wingett and Andrews, 2018). *De novo* assemblies were produced using SPAdes v3.10.1 (Bankevich et al., 2012).

### Determination of Average Nucleotide Identity (ANI) and *in silico* DNA–DNA Hybridization (DDH)

A set of 69 *Helicobacter* species reference genomes was constructed based on75 strains from the CNRCH collection (Ménard et al., 2016) (Supplementary Table S1). Five strains were removed due to missing whole genome sequencing (WGS) (*Helicobacter canadensis* NCTC 13242, *Helicobacter hepaticus* Hh-2, *Helicobacter mastomyrinus* MIT 97-5574 and MIT 94-022, and *Helicobacter nemestrinae* ATCC 49396) and one for further identification (*Helicobacter* species CNRCH 2013/518). Accession numbers for each genome and genes are available on Supplementary Table S2. ANI and DNA–DNA hybridization (DDH) values were assessed *in silico* using online tools based on assembled genomes of each of our three strains. ANI analyses were performed using the FastANI 1.1 tool. Pairwise comparisons were calculated on all 72 genomes (69 reference genomes and the 3 strains studied) using a kmer-size of 16 and a fragment length of 750^4^ (Jain et al., 2018).

DNA***–***DNA hybridization analyses were performed using a Genome-to-Genome Distance Calculator (GGDC)^5^ with the recommended local alignment tool BLAST + to compare each strain with representative genomes from the closest species identified in *gyrA*/16S rRNA-based phylogeny. A significant probability (>95%) for DDH being > 70% is applied to conclude that two strains belong to a same species.

### Genome Analyses

All of the genomic sequences and associated information were stored in a web-based Bacterial Isolate Genomic Sequence database (BIGSdb^6^) (Jolley and Maiden, 2010).

The BLAST algorithm implemented in BIGSdb was used to perform gene-by-gene alignments on the three potential novel species and representative genomes from existing *Helicobacter* species. These analyses were run independently for each potential novel species using the list of all genes from this specific species as a reference list for the alignments.

A phylogenetic tree based on *gyrA* was built from 72 nucleotide sequences from the three potential novel species and representative genomes (Ménard et al., 2016) from existing *Helicobacter* species using Molecular Evolutionary Genetics Analysis (MEGA) X software (Tamura et al., 2013). The evolutionary history was inferred using the neighbor-joining method. The percentage of replicate trees in which the associated taxa clustered together in the bootstrap test (1,000 replicates) is shown next to the branches. This analysis was repeated on the 16S rRNA gene using the same 72 species. Investigation of specific genes was carried out using alignment tools available in MEGA X.

### Colonization of Strain Cn23e in Mice

For infection in mice, the Cn23e strain was grown on MH10% agar plates and collected in brucella broth medium. Six-week-old C57BL6 mice (*n* = 5) were fasted to facilitate bacterial colonization and then force-fed for three consecutive days with a dose of around 10^8^ CFU/mouse. A control group of five non-infected mice that received brucella broth medium only, was also constituted. All experiments were performed in specific pathogen-free animal facilities at the University of Bordeaux. Only female neonates were used for experiments.

#### Histologic Experiments

Mice were euthanized at 5 weeks post-oral gavage. Half of the stomach was fixed in formaldehyde. Sections (3-mm thick) from paraffin-embedded tissues were processed for hematoxylin and eosin (H&E) staining. H&E-stained sections were coded and examined blindly by a pathologist Prof. P. Dubus, University of Bordeaux for the presence of inflammation and lymphoid infiltrates. These features were graded on a 0 to 4 or 0 to 3 point scale, respectively, as previously described (Varon et al., 2012; Chrisment et al., 2014). All slides were mounted with Eukitt mounting medium (Labonord; VWR International, Fontenay-sous-Bois, France). Slides were scanned using a digital slide scanner (Panoramic SCAN; 3DHISTECH, Ltd., Budapest, Hungary) equipped with a Zeiss objective (Plan-Apochromat 40; numerical aperture, 0.95; Carl Zeiss Microscopy GmbH, Jena, Germany) and a high-resolution color camera (VCCFC60FR19CL, 4MP; CIS Corporation, Tokyo, Japan) available at the Experimental Histopathology Platform, US 005 UMS 3427-TBM CORE. The images were read using the Panoramic Viewer software version 1.15.4 (3DHISTECH, Ltd.).

#### DNA Extraction and Quantitative PCR to Determine the *Helicobacter felis* Strain Cn23e Load in Gastric Biopsies

A quantitative PCR using Fluorescence Resonance Energy Transfer technology targeting DNA coding for *Helicobacter felis fla*A was used as previously described (Floch et al., 2017). The final results were expressed as a ratio of bacteria/murine cells. DNA extracted from the m-ICcl2 murine epithelial cell line available in the laboratory was used to express results as a bacteria/murine cell ratio. The method**’**s detection limit is approximately 0.002 bacteria/murine cells for *H. felis* as previously described (Floch et al., 2017).

### Statistical Analyses

Statistical analyses were performed with GraphPad Prism 5.01 (GraphPad Software, Inc., San Diego, CA, United States). The Mann–Whitney test was used as a non-parametric test to determine whether strain Cn23e induced a significant gastric inflammation in infected *versus* non-infected animals. Differences were considered significant when *p* was inferior to 0.05.

### Ethics Statement

This study was carried out in accordance with the principles of the Basel Declaration and recommendations of the European Union (European Directive 2010/63/EU) on animal experimentation. The project was evaluated by the local ethical committee of the University of Bordeaux and conformed to the French Ministry of Agriculture Guidelines on Animal Care and the French Committee of Genetic Engineering, with respect to the principle of the 3 Rs (replacement, reduction, and refinement). The project received the approval number A13846.

The informed consent for the use of privately owned animals was obtained from the owner, Dr. A. Touzla from the Aquivet Veterinary Clinic^7^.

No informed consent for using human helicobacter isolates were requested from the patients. Therefore, to ensure subject anonymity, all indirectly identifiable patient data were removed from the present study.

Strains obtained from Cochin Hospital (Paris, France) and Orléans Hospital (Orléans, France) were also used. Their hospital administration did not require a study review or approval by an ethics committee because the strains were sent to the French CNRCH for research purposes only.

All strains described in this study, whether of animal or human origin, will be anonymized and transferred to the Center of Biological Resources (CRB) at the Bordeaux University hospital^8^. A Material Transfer Agreement was signed between the CRB and the CNRCH^9^.

## Results

### Clinical Data

Strain CNRCH 2005/566H was isolated in 2005 from the feces of a 55–60-year old patient suffering from hepatocellular carcinoma and gastroenteritis at the Orléans Hospital, France. Strain 48519 was isolated in 2017 from the blood sample of a 25–35-year old patient, at Cochin Hospital in Paris, France. A bacteremia was detected following fever, shivers, abdominal pain, and non-bloody mucous diarrhea symptoms. Strain Cn23e was isolated in 2017 from a gastric biopsy obtained from a dog suffering from chronic gastritis hospitalized in the Aquivet Veterinary Clinic^7^ (Eysines, France).

### Biochemical and Growth Characteristics

For all three species, bacterial cells were motile, curved and Gram-negative, with a translucid and shiny aspect, evoking a helicobacter. There was no visible growth in a CO_2_ enriched or anaerobic atmosphere. Bacterial cells underwent transformation to coccoidal forms upon exposure to air and after prolonged incubation (data not shown).

CNRCH 2005/566H colonies were visible on trypticase soy agar plates at 35, 37, or 42°C after 24 h or at 30°C after 48 h under microaerobic conditions (Supplementary Figure S1A). Catalase and urease activity was detected, but not γ-glutamyl transpeptidase activity. The API^®^ Campy strip showed that strain CNRCH 2005/566H was positive for nitrate reduction and alkaline phosphate hydrolysis. According to antibiotic susceptibility testing, CNRCH 2005/566H was susceptible to levofloxacin, clarithromycin, tetracycline and metronidazole, but resistant to cephalothin, rifampicin, and amoxicillin. These characteristics were unique to strain CNRCH 2005/566H (Table 1).

**TABLE 1:** Phenotypic characteristics that differentiate “*H. labetoulli* sp. Nov.” strain CNRCH 2005/566H and *“H. burdigaliensis* sp. Nov.” strain 48519 and *H. felis* strain Cn23e from other *Helicobacter* species.

**Characteristic**	**CNRCH 2005/566H**	**48519**	**Cn23e**	**4**	**5**	**6**	**7**	**8**	**9**	**10**	**11**	**12**	**13**	**14**	**I5**	**16**	**17**	**18**	**19**	**20**	**21**	**22**	**23**	**24**	**25**	**26**	**27**	**28**	**29**
Catalase production	+ g	+ g	+ g	+	+	+	+	+	+	+	(+)	(+)	+	+	+	+	+	(+)	+	+	+	+	+	+	+	(−)	+	+	+
Nitrate reduction	+	+	+	+	+	+	v	−	+	+	−	+	−	−	−	−	+	+	+	+	+	+	+	+	(+)	+	−	v	+
Urease	+ g	−	+ g	−	−	−	−	+	+	(+)	−	−	+	+	+	−	+	−	+	(+)	+	−	−	+	−	−	+	−	+
Alkaline phosphate hydrolysis	+	−	+	+	+	−	−	+	+	v	(−)	(−)	+	+	+	+	−	(+)	−	v	−	+	−	v	+	−	−	−	+
Gamma-glutamyl transpeptidase	−	−	+ g	−	−	−	−	+	+	+	−	−	ND	+	+	+	−	−	+	+	+	−	−	+	−	ND	+	−	+
Growth at 42°C	+	−	−	(−)	+	+	v	+	+	+	+	v	−	−	v	(−)	−	−	+	−	v	+	+	+	(−)	+	−	+	+
Susceptibility to:																													
Nalidixic acid (30 μ*g)*	I	R	S	R	S	R	R	R	R	S	I	S	ND	R	R	S	S	R	S	R	S	R	S	R	R	R	R	R	S
Cephalothin (30 μ*g)*	R	I	S	S	R	R	S	R	R	I	R	I	ND	R	S	S	R	R	R	R	R	S	S	S	S	R	S	R	R
No. flagella per cell	2	2	15–20	2–5	7–10	3–14	10–20	2	1–2	2	1	1–2	6–12	1	14–20	2	2	2	2	10–14	4–8	4–8	2	1	4–8	2	10–23	5–7	2
Sheathed flagella	No	Yes	Yes	Yes	Yes	Yes	Yes	Yes	No	Yes	Yes	Yes	Yes	Yes	Yes	Yes	No	Yes	No	Yes	Yes	Yes	Yes	No	Yes	No	Yes	Yes	Yes
Distribution of flagella	B	B	B	B	B	B	B	B	B	B	M	B	B	M	B	B	B	B	B	B	P	B	B	M	B	B	B	B	B

*Data were obtained from this study and published studies (Dewhirst et al., 2000; Fox et al., 2000; Patterson et al., 2000; Simmons et al., 2000; Franklin et al., 2001; Robertson et al., 2001; Van den Bulck et al., 2006; Moyaert et al., 2007; van der Mee-Marquet et al., 2017).Taxa: (1) “*H. burdigaliensis* sp. nov.” CNRCH 2005/566H; (2) “*H. labetoulli* sp. nov.” 48519; (3) *H. felis* Cn23e; (4) *H. caesarodunensis*; (5) *H. equorum*; (6) *H. pullorum*; (7) *H. canadensis*; (8) *H. pylori*; (9) *H. mustelae*; (10) *H. felis*; (11) *H. fennelliae*; (12) *H. cinaedi*; (13) *H. nemestrinae*; (14) *H. muridarum*; (15) *H. acinonychis*; (16) *H. canis*; (17) *H. hepaticus*; (18) *H. pametensis*; (19) *H. bilis*; (20) *H. bizzozeronii*; (21) *H. trogontum*; (22) *H. cholecystus*; (23) *H. rodentium*; (24) *H. salomonis*; (25) *H. mesocricetorum*; (26) *H. ganmani*; (27) *H. aurati*; (28) *H. typhlonius*; (29) *H. cynogastricus*. +, 100% positive strains; −, 100% negative strains; (+), 80–94% positive strains; v, 42–66% positive strains; (−), 7–33% positive strains; g, gene(s) linked to this phenotypic characteristic found in the genome; S, sensitive; R, resistant; I, intermediate; ND, not determined; A, amphitrichous; B, bipolar; M, monopolar; P, peritrichous.*

Colonies from strain 48519 (Supplementary Figure S1B) were visible on trypticase soy agar plates or MH10% agar plates at 35°C after 24 h under microaerobic conditions. Catalase activity was observed, but not urease or γ-glutamyl transpeptidase activities. API^®^ Campy strip showed that strain 48519 was positive for nitrate reduction but not for alkaline phosphate hydrolysis. According to antimicrobial susceptibility testing, strain 48519 was susceptible to rifampicin, tetracycline and metronidazole, but resistant to nalidixic acid, levofloxacin, clarithromycin, and amoxicillin. These characteristics were close to those of *Helicobacter cinaedi* (Table 1).

Colonies of strain Cn23e (Supplementary Figure S1C) were visible on MH10% agar plates at 35°C after 48 h under microaerobic conditions. Catalase, urease, γ-glutamyl transpeptidase and hippuricase activity was observed. The API^®^ Campy strip showed that Cn23e were positive for nitrate reduction but not for alkaline phosphate hydrolysis. According to antimicrobial susceptibility testing, Cn23e was susceptible to all antibiotics tested. Cn23e characteristics were similar to those of *H. felis* (Table 1).

### Morphological Characteristics

**FIGURE 1:**
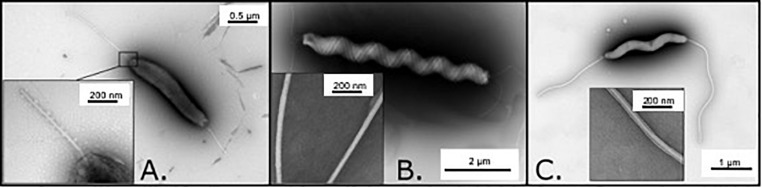
Observation of the three investigated isolates by transmission electron microscopy. **(A)** Observation of CNRCH 2005/566H revealing an amphitrichous bacterium with unsheathed flagella. Length of the bacteria (flagella excluded) was 2 μm on average. **(B)** Observation of Cn23e revealing a lophotrichous bacterium with sheathed flagella. Length of the bacteria (flagella excluded) was 6 μm on average. **(C)** Observation of 48519 revealing an amphitrichous bacterium with sheathed flagella. Length of the bacteria (flagella excluded) was 4 μm on average.

Microscopic observation of CNRCH 2005/566H revealed a rod-shaped bacterium, approximately 2 μm long and 0.3 μm wide (Figure 1A). Two amphitrichous unsheathed flagella with a diameter of around 20 nm were visible.

Microscopic observation of Cn23e revealed a tightly wound spiral bacterium, approximately 6 μm long and 0.5 μm wide (Figure 1B). A high number (>10) of sheathed flagella with a diameter of around 30 nm were observed at both extremities of the cell.

Microscopic observation of strain 48519 revealed a tightly wound spiral bacterium, approximately 4 μm long and 0.25 μm wide (Figure 1C). Two amphitrichous sheathed flagella with a diameter of around 40 nm were visible.

### Phylogeny

Two phylogenetic analyses were performed: using the *gyr*A and 16S rRNA genes. *gyr*A based phylogeny has been shown to better discriminate among species of the *Helicobacter* genus (Ménard et al., 2016) whereas 16S rRNA is known to provide imprecise results (Dewhirst et al., 2005). The *gyrA* sequences used in the present study correspond to those previously described by Ménard et al. (2016). They are representative of the main *Helicobacter* species described (and/or published) in humans and animals either for the gastric helicobacters and the enterohepatic helicobacters.

**FIGURE 2:**
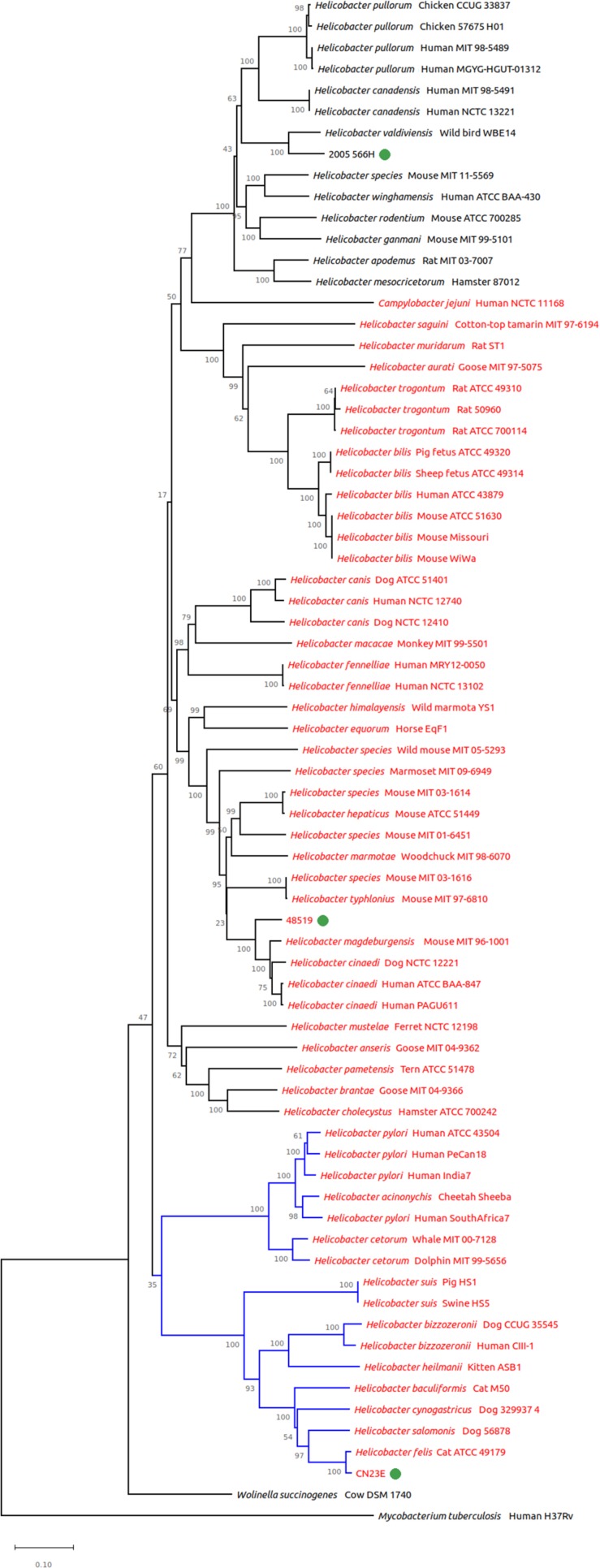
Genomic tree from *gyr*A based phylogeny built with 72 nucleotide sequences. The evolutionary history was inferred using the neighbor-joining method. The proportion of replicate trees in which the associated taxa clustered together in the bootstrap test (1,000 replicates) is shown next to the branches. Branches in blue correspond to gastric species, branches in black to enterohepatic species. *Helicobacter* species named in red are sheathed helicobacters, those named in black are unsheathed species.

In this study, *gyr*A phylogeny placed the two potential novel species among the enterohepatic species (Figure 2). CNRCH 2005/566H clustered with *Helicobacter valdiviensis*, among unsheathed helicobacters, with a notable distance between these two species. Strain 48519 clustered closely with *H. cinaedi* and ‘*Helicobacter magdeburgensis’* (sheathed helicobacter). Cn23e clustered closely with *H. felis*, among the gastric species.

**FIGURE 3:**
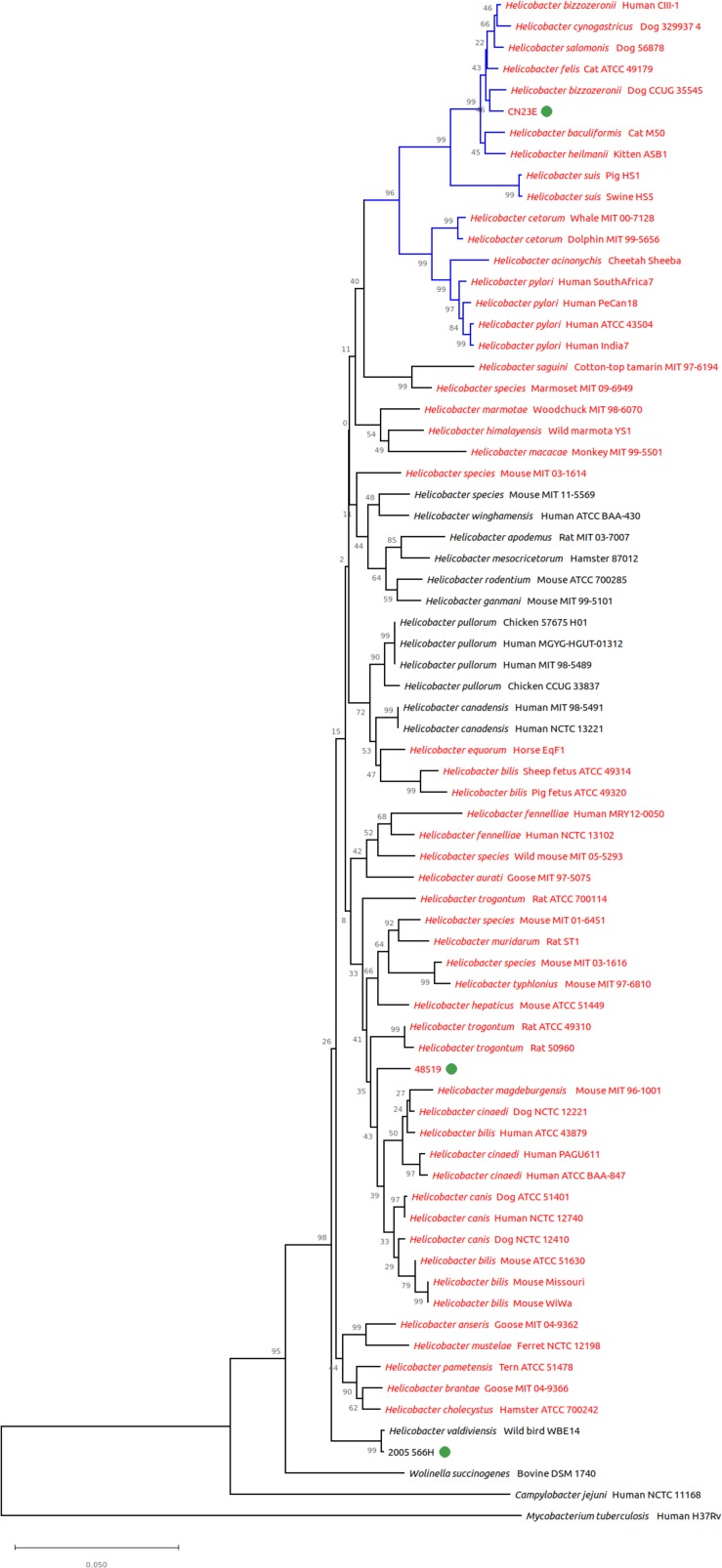
Genomic tree from 16S rRNA gene based phylogeny built with 72 nucleotide sequences. The evolutionary history was inferred using the neighbor-joining method. The proportion of replicate trees in which the associated taxa clustered together in the bootstrap test (1,000 replicates) is shown next to the branches. Branches in blue correspond to gastric species, branches in black to enterohepatic species. *Helicobacter* species named in red are sheathed helicobacters, those named in black are unsheathed species.

16S rRNA gene based phylogeny clustered all three studied species among the same groups (Figure 3). CNRCH 2005/566H was also reliably positioned close to *H. valdiviensis*. However, strain 48519 diverged from other near sheathed helicobacters and Cn23e no longer clustered closely with *H. felis* but with *H. bizzozeronii*.

### Taxonomy

The two best ANI and DDH scores for each strain are indicated in Table 2. Supplementary Data are provided in Supplementary Table S1.

**TABLE 2:** Two best average nucleotide identity (ANI) and pairwise *in silico* DNA–DNA hybridization (DDH) scores for strains CNRCH 2005/566H, 48519 and Cn23e.

**Query genome**	**Reference genome**	**ANI**	**Formula 1**			**Formula 2**			**Formula 3**				**G+C difference**
			**DDH**	**Model C.I.**	**Distance Prob. DDH ≥ 70%**	**DDH**	**Model C.I.**	**Distance Prob. DDH ≥ 70%**	**DDH**	**Model C.I.**	**Distance**	**Prob. DDH > = 70%**	
**CNRCH 2005/566H**	*H. valvidiensis WBE14*	84.97	34.20	[30.9–37.8%]	0.4559 0.36	30.50	[28.1–33%]	0.1394 0.13	32.40	[29.4–35.4%]	0.5318	0.01	1.69
**CNRCH 2005/566H**	*H. canadensis MIT 98-5491*	76.86	13.90	[11.1–17.3%]	0.9264 0	20.50	[18.3–22.9%]	0.2141 0	14.20	[11.8–17%]	0.9421	0	0.19
**48519**	*H. cinaedi PAGU611*	89.71	47.4000	[44–50.8%]	0.3268 7.92	40.40	[37.9–42.9%]	0.0981 3	45.60	[42.6–48.7]	0.3929	0.83	0.39
**48519**	*H. cinaedi ATCC BAA-847*	89.70	45.60	[42 2–49%]	0.3418 5.63	40.50	[38.1–43 1]	0.0976 3.11	44 10	[41.1–47.1%]	0.4060	0.53	0.68
**Cn23e**	*H. felis ATCC 49179*	97.40	88.00	[84 5–90.8%]	0.0915 96.51	7520	[72 2–78%]	0.0292 85.97	88.70	[85 8–91%]	0.1181	99.21	0.41
**Cn23e**	*H. cynogastricus strain 329937_4*	86.29	30.50	[27.2–34.1%]	0.5051 0.11	35.50	[33.1–38%]	0.1160 0.78	30.10	[27.2–33.2%]	0.5625	0	0.84

*ANI analyses were performed using FastANI 1.1 tool (see section “Materials and Methods”). The DDH value were calculated using Genome-to-Genome Distance Calculator (v. 2.1; http://ggdc.dsmz.de/distcalc2.php) with the three formulas: formula: 1 (HSP length/total length); formula: 2 (identities/HSP length); formula: 3 (identities/total length).HSP, high-scoring segment pairs.*

ANI analyses were performed to measure nucleotide-level genomic similarity among all pairs of previously selected genomes, including each potential novel species. It reveals that two of them, CNRCH 2005/566H and 48519, showed ANI percentages lower than the speciation threshold (95%) (Klappenbach et al., 2007) when compared with genomes from the public database. More specifically, the closest species to CNRCH 2005/566H was *H. valdiviensis* with a non-significant ANI value of 84.9%. Three of the closest species to 48519 belong to *H. cinaedi species*, with ANI values of 88.8%, 89.7% and 89.7%. This is lower than the speciation threshold, suggesting the closeness of this strain with the *H. cinaedi* species but indicating the occurrence of a potentially new species. On the other hand, Cn23e was confirmed to belong to the species *H. felis*, with ANI value of 97.4%.

DNA***–***DNA hybridization analyses confirmed the ANI results. For strain CNRCH 2005/566H, all DDH scores were much lower than 40%. For strain 48519, DDH scores were lower compared to CNRCH 2005/566H with an average of 15%. Finally DDH analyses confirmed that Cn23e belongs to the *H. felis* species, with a score of 75.2%.

### Genome Content

The CNRCH 2005/566H genome consisted of 1,803,884 bp, a GC% of 32.8, and 1,867 coding sequences (RAST annotation). The strain 48519 genome consisted of 2,092,710 bp, a GC% of 38.6, and 2,335 coding sequences (RAST annotation). The Cn23e genome consisted of 1,638,090 bp, a GC% of 44.7, and 1,718 coding sequences (RAST annotation).

An analysis of the number of genes shared by each of the three strains with representative genomes of validated species revealed that 94.2% of the genes present in Cn23e were also present in *H. felis* strain ATCC 49179. 48519 genome shared only 77.3% of its genes with *H. cinaedi* strain ATCC BAA-847. CNRCH 2005/566H genome shared only 48.9% of its genes with *H. pullorum* strain MIT 98-5489 and 47.9% with *H. canadensis* strain MIT 98-5491.

Biochemical characteristics described earlier were confirmed in the genome (Table 1). RAST annotation revealed the presence of the two catalase subunits and a large number of urease subunits present in one unique copy in the CNRCH 2005/566H genome. Genes coding for RTX (hemolysin) and elements linked to type II secretion systems were also present. The two catalase subunits were also present in strain 48519, but urease units were missing. Genes coding for elements linked to type II secretion systems were present. The presence of a nitrate reductase operon, two genes associated with nitrates, and three coding sequences annotated as esterases were also detected.

The genome content of Cn23e was close to the *H. felis* reference strain CS1 (Lee et al., 1990). The presence of the gene coding for hippuricase was highlighted, and had not been described in *H. felis* until now. The presence of genes coding for hippuricase was verified by tBLASTn analysis in the published *H. felis* reference genome ATCC 49179 (FQ670179 71333-72457, locus tag HFELIS_01060) with 91% identity, and in other members of the *Helicobacter* genus, namely *H. heilmannii* (HE984298 1036772-1037920, locus tag BN341_11590) with 65% identity and *H. cinaedi* (AP012492 1742369-1743523, locus tag HCBAA847_1843) with 26% identity, despite the absence of literature concerning this point.

#### Infection Experiments on Mice

**FIGURE 4:**
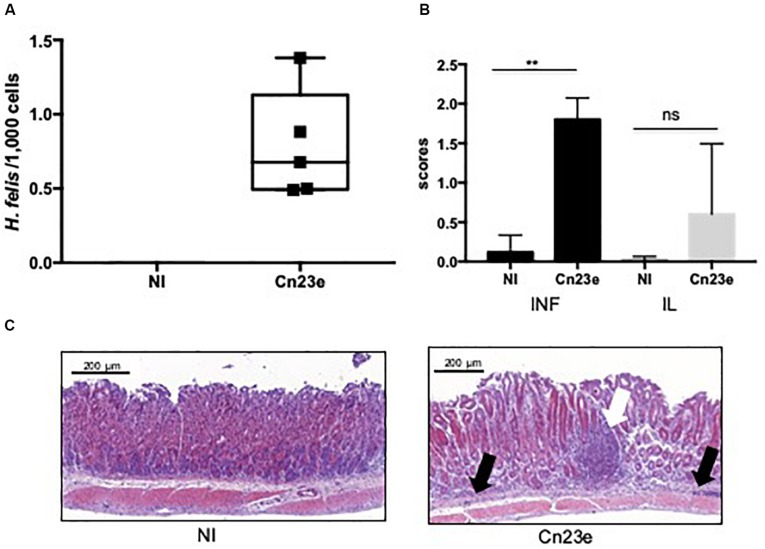
Histological analyses of Cn23e-infected C56BL6 mice at 6 weeks post-oral gavage. **(A)** Quantification of the bacterial load in gastric biopsies from infected mice. Results were obtained by quantitative PCR as described in Section “Materials and Methods.” *H. felis*/1,000 murine cell ratio in non-infected (NI) and Cn23e-infected mice at 6 weeks post-infection (*n* = 5) versus 18 months post-infection (*n* = 5 for each group). Graphic representations are box plots, with the box representing 50% of values around the median (horizontal line) and the whiskers representing the minimum and maximum figures of all of the data. ^∗∗^*p* < 0.01, ns, non-significant. **(B)** Inflammation (INF), lymphoid infiltration (IL) scoring: for inflammation, scores range from 0 to 4; for the lymphoid infiltration, scores range from 0 to 3 (see section “Materials and Methods”). Data are plotted as bar graphs displaying the median ± standard deviation for each group. Non-infected (NI) (*n* = 5), *Cn23e*-infected (*n* = 5). For each group, the bar represents the arithmetic median of scores. ^∗∗^*p* < 0.01, ns, non-significant for Cn23e-infected mice versus NI. **(C)** Sections (3 μm thick) from paraffin-embedded tissues were processed for H&E staining. Partial sections of the large curvature of the stomach (fundus and corpus) are presented on these pictures. Representative histopathological features of NI and Cn23e-infected mice: polymorphonuclear infiltrates (black arrow) and lymphoid infiltrates (white arrow) are shown. Scale bars: 200 μm.

At 6 weeks post-oral gavage Cn23e-was detected by PCR in all infected mice. The level of colonization estimated by qRT-PCR was 0.8 *Helicobacter*/1,000 cells (Figure 4A). Significant leukocyte infiltration and a few scattered small lymphoid infiltrates were observed in the stomachs of the Cne23-infected mice (Figures 4B,C). Leukocyte infiltration present at the base of the mucosa was composed of polymorphonuclear cells. Lymphocytes were also observed within the gastric mucosa alongside glands or organized in small lymphoid aggregates, an aspect compatible with a lymphocytic gastritis. There was no sign of inflammation in non-infected mice (Figures 4B,C).

## Discussion

Among the three isolates that were studied in the present article, two may correspond to two new *Helicobacter* species. The third one, strain Cn23e, is a new isolate of *H. felis*.

The first species proposed in the present study is ‘*Helicobacter burdigaliensis* sp. nov.’ ‘Burdigaliensis’ refers to the latin name of Bordeaux where the proposed type strain, CNRCH 2005/566H, was identified. This data has been submitted to GenBank under the accession number QXJG00000000. The version described in this paper is version QXJG00000000.1. Raw reads are available at SRA under the accession number SRS3744858.

The second species is ‘*Helicobacter labetoulli* sp. nov.’ ‘Labetoulli’ refers to the family name of the microbiologist who isolated the proposed type strain 48519.

Finally, strain Cn23e is a new member of the *H. felis* species: this new *H. felis* isolate could be an alternative choice to the extensively used *H. felis* CS1 strain for long term *in vivo* experiments in mice (Lee et al., 1990). It has a natural capacity to colonize mouse gastric mucosa and was submitted to minimal *in vitro* subculturing.

This work reflects the difficulties in identifying new species of helicobacters by using phenotypic methods only. Indeed, helicobacters are able to colonize alternative sites of the digestive tract (saliva, stomach, cecum, colon, liver) in various hosts (mammals, birds, reptiles) leading to constant adaptation of *Helicobacteraceae* to novel niches over time. These adaptation capacities are reflected in gene acquisition and divergent gene evolution and constitute the main obstacle in determining helicobacter taxonomy and phylogeny. Thus genome sequencing and bioinformatics are valuable tools to support *Helicobacteraceae* taxonomy.

## Conclusion

The *Helicobacteraceae* family obviously has not yet revealed all of its secrets.

## Data Availability Statement

The datasets generated for this study can be found in the GenBank-Accession Numbers: QXJG00000000, QXJF00000000, and QXJE00000000. SRA-accession numbers: SRS3744858, SRS3744857, and SRS3736525.

## Ethics Statement

The animal study was reviewed and approved by University of Bordeaux; approval number A13846.

## Author Contributions

The manuscript was written by ElB, with help from QJ, AM, CV, LB, FM, EmB, and PL. JR isolated the strain 48519 and collected patient related information. OB, AD, and AB helped characterize the CNRCH 2015/518H strain. ElB and QJ performed the bioinformatic analyses. LB, SL, and EG were in charge of the imaging. AG performed the *in vivo* analysis. OT provided the strain Cn23e. All authors reviewed the manuscript.

## Conflict of Interest

The authors declare that the research was conducted in the absence of any commercial or financial relationships that could be construed as a potential conflict of interest.

## Footnotes

1 http://www.bacterio.net/helicobacter.html

2 https://www.sfm-microbiologie.org/2019/05/06/casfm-eucast-2019-v2/

3 www.helixio.com

4 https://github.com/ParBLiSS/FastANI

5 http://ggdc.dsmz.de/ggdc.php#

6 http://zoo-dalmore.zoo.ox.ac.uk/

7 http://www.aquivet.fr

8 https://www.chu-bordeaux.fr/Professionnels-recherche/Centre-de-Ressources-Biologiques/

9 www.cnrch.fr

## Supplementary Material

FIGURE S1 Aspect of the colonies of the three investigated isolates. **(A)** Strain 2005/566H; **(B)** strain 48519, after 72 h on trypticase soy agar plates; **(C)** strain Cn23e after 48 h on Mueller Hinton (10% sheep blood) agar plates. All plates were incubated at 35°C under microaerobic conditions.

TABLE S1 Average nucleotide identity based on BLAST (ANIb) and pairwise *in silico* DNA–DNA hybridization (DDH) values (%) for strains CNRCH 2005/566H, 48519 and Cn23e and other *Helicobacter* species. The DDH values were calculated using Genome-to-Genome Distance Calculator (v. 2.1; http://ggdc.dsmz.de/distcalc2.php) formula 2 (isDDH estimates based on identities/HSP length). The *Helicobacter* species and accession numbers for each genome and genes used for the analyses are indicated on the second sheet of the Table. HSP, high-scoring segment pairs.

TABLE S2 Strain list used in the study. WGS, 16S rRNA gene, and *gyrA* accession numbers are indicated. Similar accession numbers means that the nucleotide sequences of 16S rRNA and *gyrA* genes were extracted from Genbank annotation or with blastn command line tool (using WGS file as “subject” and the 16S rRNA or *gyrA* sequence from another strain as “query”).
