# Supplementary figures and images for: Whole-Genome Sequencing and Bioinformatics as Pertinent Tools to Support Helicobacteracae Taxonomy, Based on Three Strains Suspected to Belong to Novel Helicobacter Species
Source: Front Microbiol. 2019 Dec 6;10:2820. doi: 10.3389/fmicb.2019.02820 (PMC6908825; doi:10.3389/fmicb.2019.02820)

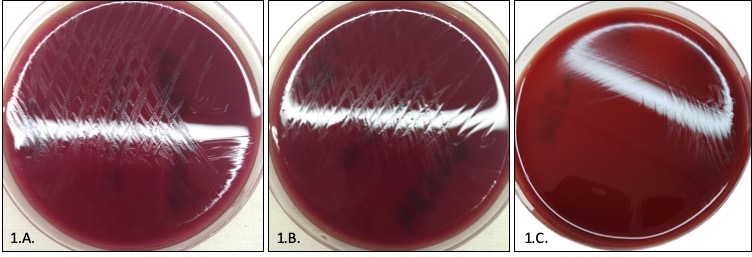

Supplement: FIGURE S1 — Aspect of the colonies of the three investigated isolates. (A) Strain 2005/566H; (B) strain 48519, after 72 h on trypticase soy agar plates; (C) strain Cn23e after 48 h on Mueller Hinton (10% sheep blood) agar plates. All plates were incubated at 35°C under microaerobic conditions. [file Image_1.JPEG]
